# Emerging Polymer Materials in Trackable Endovascular Embolization and Cell Delivery: From Hype to Hope

**DOI:** 10.3390/biomimetics7020077

**Published:** 2022-06-10

**Authors:** Md Mohosin Rana, Marites P. Melancon

**Affiliations:** 1 Biomedical Engineering Graduate Program, Schulich School of Engineering, University of Calgary, Calgary, AB T2N 1N4, Canada; 2 Department of Interventional Radiology, The University of Texas MD Anderson Cancer Center, Houston, TX 77030, USA; mmelancon@mdanderson.org

**Keywords:** polymers, embolic agents, hepatocellular carcinoma, uterine fibroids, arteriovenous malformations, embolization

## Abstract

Minimally invasive endovascular embolization is a widely used clinical technique used for the occlusion of blood vessels to treat various diseases. Different occlusive agents ranging from gelatin foam to synthetic polymers such as poly(vinyl alcohol) (PVA) have been commercially used for embolization. However, these agents have some drawbacks, such as undesired toxicity and unintended and uncontrolled occlusion. To overcome these issues, several polymer-based embolic systems are under investigation including biocompatible and biodegradable microspheres, gelling liquid embolic with controlled occlusive features, and trackable microspheres with enhanced safety profiles. This review aims to summarize recent advances in current and emerging polymeric materials as embolization agents with varying material architectures. Furthermore, this review also explores the potential of combining injectable embolic agents and cell therapy to achieve more effective embolization with the promise of outstanding results in treating various devastating diseases. Finally, limitations and challenges in developing next-generation multifunctional embolic agents are discussed to promote advancement in this emerging field.

## 1. Background

Vascular intervention therapies (VITs) are minimally invasive strategies that have been developed over the last few decades to replace complex surgery for the treatment of vascular malformation, cancer, and hemorrhage control [1,2]. These strategies drew attention recently due to their minimally invasive and image-guided interventions which often result in better outcomes with fewer complications [3]. Blood vessels are considered common routes of entry into the body in any endovascular approach. Embolization is a VIT where blood flows are deliberately blocked around the blood vessels of a target lesion via intravascular deposition of embolic agents from catheters for therapeutic purposes [4]. Previous reports demonstrated that therapeutic endovascular embolization has the potential to treat acute gastrointestinal bleeding, arteriovenous malformations, fistulas, and targeted cancer treatments [5,6,7,8].

In the past, autologous blood clots, muscle fragments, or stainless-steel pellets were used in embolization [9]. In the 1970s, due to the failure of natural analogues for embolization, researchers advanced to drive the development of modern materials as embolic agents, i.e., gelatin sponges [10]. Gelatin sponges were first used to occlude a carotid-cavernous fistula and showed successful occlusion without any vision loss or complications in the patient after surgery [10].

Embolic agents are broadly two types, i.e., mechanical embolic agents such as metal coils and plugs and flow-directed embolic agents such as polymers, particulates, or in situ gelling materials [9]. The mechanical embolic agents are not within the scope of this review. A detailed review of this topic can be found elsewhere [11]. Flow-directed agents can be delivered via catheters positioned within a specific vascular supply to treat vascular malformations. From a clinical standpoint, depending on the therapeutic goal, flow-directed agents are subdivided into temporary and permanent embolic agents [12]. A temporary fast-acting embolic agent is required to obstruct a hemorrhaging vessel, while embolization for a vascular malformation often requires a more precise and permanent embolic material. A wide range of calibrated microspheres and bioglue made of both natural and synthetic polymers have been developed as flow-directed embolics [4]. Although these materials have advantages, they also come with shortcomings that need to be resolved to provide a unique materials platform for embolization. Biocompatibility, biodegradability, ease of delivery, trackability, resistance to migration, and the ability to act as a therapeutic delivery vehicle are some general properties of an attractive embolic agent [13,14]. Unfortunately, none of the existing embolic agents are ideal for these properties. For example, a gelatin sponge made from purified porcine skin comes in the form of dry sheets and is commonly used as a temporary occlusive material [15,16]. This material provides a rapid, low-cost, and transient embolic in various embolization procedures. However, this material has pitfalls such as lack of precision in delivery and unpredictable embolization that hinders its application [17]. Poly(vinyl alcohol) or PVA-based calibrated microspheres have been designed to resolve this issue and provide better embolization [18]. Despite the highly predictable level of embolization of these materials, nondegradability is still an utmost concern that needs attention. Degradable polymeric embolics consisting of recombinant silk-elastin-like block copolymers or chitosan derivatives have been developed to address these issues [19]. Furthermore, drugs and other biological agents have been loaded onto these embolic materials to enhance their efficacy. For example, current minimally invasive therapy for both hepatocellular carcinoma (HCC) and aneurysmal subarachnoid hemorrhage (aSAH) utilizes endovascular embolization to starve the cells in tumors (in HCC) or block the blood flow to prevent rupture (in aSAH) via arterial occlusion [3,20]. Stem cell therapy can be an add-on with embolization to reduce the inflammation and induce damaged tissue repair in HCC and aSAH treatment. Therefore, the combination of trackable polymeric embolics and cell therapy may provide promising outcomes in treating these devastating disease conditions.

The main objective of this review is to provide a comprehensive and critical evaluation of recent advances in polymeric embolic agents for endovascular embolization and stem cell delivery. The current progress in emerging polymer materials for embolization were searched for on Google Scholar, Web of Science, and PubMed using the keywords “polymer embolic agent”, “embolization”, and “arteriovenous malformations”. Initially, the embolization techniques and the clinical needs of these techniques are highlighted. Then, a series of conventional and emerging polymeric embolics with their physical properties, advantages, and disadvantages are comprehensively reviewed. A brief detail on the polymeric embolic agents with trackability/imaging ability is described, followed by a discussion of potential polymer-based carriers for stem cell delivery. Finally, current limitations and future directions in the polymer-based embolic agents for embolization and cell delivery are critically explored.

## 2. Endovascular Embolization

### 2.1. Clinical Applications

Currently, embolization is performed in different clinical scenarios. The main goal of embolization is to block blood flow; hence, successful embolization occurs when hemostasis is accomplished. Examples of clinical complications treated with embolization include hemorrhage, arteriovenous malformation (AVM), aneurysms, and hypervascular solid tumors (Table 1).

A hemorrhage is a fatal condition that occurs due to trauma, underlying medical condition, or both [21]. Blood escapes the circulatory system either internally or externally in a hemorrhagic state and often leads to serious complication such as cardiogenic shock or even death (mortality rate around 40%) [22]. Hemorrhages can occur anywhere in the body, including the head, lungs, urinary tract, gastrointestinal tract, or reproductive organs [23]. In addition to conventional open surgery, another option is transcatheter arterial embolization (TAE), which provides rapid and effective hemorrhage control using a minimally invasive approach [24]. For example, the overall procedural and clinical success rates of embolization for gastrointestinal bleeding have been reported to be 93–100% and 51–88%, respectively [25]. The main goal of embolization in a hemorrhage is to precisely decrease arterial perfusion pressure and blood extravasation from the bleeding vessels. It is also important to maintain adequate collateral blood flow simultaneously to minimize ischemic injury during embolization. Moreover, a hemostatic clot may also develop to further halt the bleeding and provide the healing of injured vessel walls. However, the clot-forming ability may often compromise patients with coagulation disorders. Therefore, it is indispensable to develop an ideal embolic agent that can be used as an intrinsic plug to achieve hemostasis in coagulopathic patients.

Aneurysms are dilations of the artery due to the weakening of the vessel walls [26]. Aneurysms can occur throughout the body but most often in the cerebral circulation and peripheral arteries [27]. The walls of cerebral arteries lack a structural layer (i.e., external elastic lamina) and are present in peripheral arterial systems. As a result, their compliance reduces while their susceptibility improves to intracranial aneurysm formation. Additionally, hypertension and atherosclerosis are other risk factors for intracranial aneurysm formation [28,29]. The most effective treatment option to treat patients with brain aneurysms is endovascular therapies such as embolization. The primary goal of embolization is to fill the artery, inhibit it from circulation, and reduce the risk of arterial dilation and rupture. Designing microcatheters, coils, and embolic materials are essential in treating aneurysms by embolization [30,31]. A higher degree of precision and a wide range of available sizes are the two crucial features of the materials for embolization. The lack of these features may cause nontarget embolization, which can have significant effects on patients, such as ischemia and infarction [32]. Moreover, trackability of the materials on imaging tools is also key to monitoring the real-time deployment of the material and assessing the outcomes of the intervention in follow-up imaging [33,34].

Arteriovenous malformation (AVM) is an abnormal connection between an artery and vein which results in high-pressure shunting of arterial blood to the venous circulation directly [35]. As a result, excessive stress occurs in the venous wall and thus, overstressed veins become enlarged, stretched, and often rupture. Although AVMs can occur anywhere in the body, most typical AVMs occur in the central nervous system with high morbidity and mortality [36]. Generally, AVMs are treated by radiosurgery, microsurgery, and embolization [37]. In case of acute bleeding from an AVM, it requires urgent embolization to prevent fatal complications. Some of the common embolic agents for AVM treatment are coils, liquids, and particulates [38]. Embolization helps to return venous pressure to normal by embolizing the arteriovenous shunt. Most effective embolic agents are liquid materials such as absolute alcohol, N-butyl cyanoacrylate glues (e.g., TruFill, Cordis, Miami Lakes, FL, USA), and Onyx embolic system (Micro Therapeutics, Inc., Irvine, CA, USA) [39]. These liquid embolics homogeneously fill the vascular area and limit the risk of the secondary reopening of the embolized region (i.e., recanalization). Visibility and trackability of the embolic materials are necessary to treat AVMs effectively [4]. Moreover, embolic agents should also be deployable by microcatheters and durable to prevent recurrence and recanalization.

**Table 1:** Clinical applications of endovascular embolization using polymer embolics.

Clinical Application	Polymer Embolics for Embolization	Treatment Outcome	Future Goal	Reference
Vascular malformations (Hemorrhage, Aneurysms, AVMs)	Liquid embolics (NBCA, PHIL^TM^, Onyx^®^)	- Decrease arterial perfusion pressure - Prevention of hemorrhage - Permanent occlusion - Reduce the risk of arterial dilation and rupture (in aneurysms)	- Nonadhesive liquid embolics	[24,30,31,37]
Hypervascular tumors (e.g., hepatocellular carcinoma or HCC)	Drug eluting beads/microspheres (e.g., calibrated PVA, gelatin sponge, crosslinked starch particles)	- Starve the tumors by blocking blood flow - Local delivery of therapeutics to induce tumor necrosis - Enhance infarction	- Trackable microspheres - Degradability - Multidrug carrying ability	[40,41,42]
Uterine fibroids	Calibrated PVA microspheres	- Temporary occlusion to prevent blood loss - Induction of infarction	- Degradable microspheres	[43]

**Figure 1:**
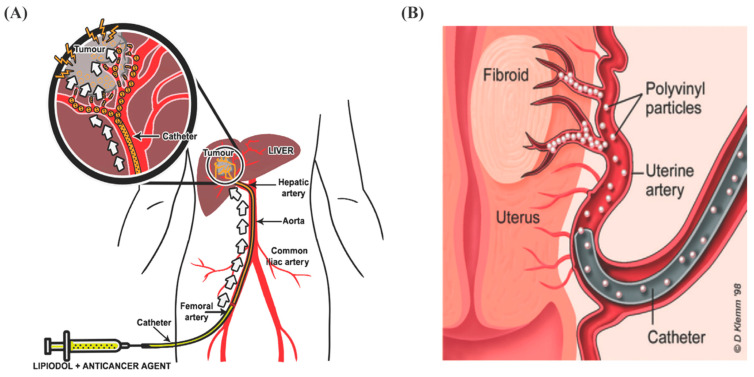
Embolization strategy for the treatment of hepatocellular carcinoma and uterine fibroid. (**A**) Injection of commercial embolic agent Lipiodol combined with anticancer therapeutic agents into the hepatic artery through catheter. Reproduced with permission from Ref. [41]. (**B**) Embolization to treat uterine fibroid by using polyvinyl particles instilled through a catheter. Reproduced with permission from Ref. [49].

Hypervascular tumors are abnormal vasculatures with increased numbers of blood vessels feeding into tumors [44]. Although surgical resection is the general strategy to control malignant tumors, the higher blood flow induces the risk of bleeding during surgery [45]. Moreover, sometimes tumors can be hard to remove due to their size, location, or patient comorbidities. HCC is the most common hypervascular tumor of the liver and is responsible for the third most common cancer-related death in the world [46]. The most effective curative options for treating HCC are surgical resection, ablation, or transplantation [47]. However, around 85% of patients are considered ineligible for such treatments during the time of diagnosis. As a result, most patients experience poor prognoses and their median survival times become less than a year. Liver tumors are unique because they receive the majority of blood from the hepatic artery, while the healthy liver parenchyma draws most of its blood from the portal system [48]. Therefore, embolization can be an option to starve the tumors by blocking blood flow from the hepatic artery, resulting in tumor shrinkage and decreasing the risk of bleeding (Figure 1A). Such a strategy will help improve surgical outcomes while preserving the surrounding hepatic tissue and the quality of patients’ life. Moreover, different embolization techniques in highly vascular tumors and resectable tumors preoperatively decrease intraoperative bleeding, which is a prime concern during surgery [40,42].

Uterine fibroids are a common type of benign tumor of the uterus [50]. Embolization shows superior efficacy for treating such benign tumors over invasive treatment such as surgery (Figure 1B). Since fibroids receive blood from uterine arteries, uterine artery embolization (UAE) can be used to block the blood flow to the uterus, resulting in shrinkage of the fibroids and a reduction in uterine bleeding [43]. Compared with conventional myomectomy and hysterectomy, UAE presents a more effective, safe, and minimally invasive strategy for fibroid management.

### 2.2. Embolization Techniques

Embolic therapies are performed to achieve hemostasis in different clinical scenarios. Transcatheter arterial embolization (TAE) is a minimally invasive approach that requires various embolic agents to block one or more blood vessels through an intravascular catheter [17]. In a typical TAE process, a catheter is used to access the target vessel, and the location is confirmed by injecting a contrast agent. Later, the same catheter delivers the selected embolic agent to prevent blood flow. Typical diseases targeted for TAE include gastrointestinal tract hemorrhage, bleeding due to vascular malformations such as arteriovenous malformations (AVMs), neoplasms such as hepatocellular carcinoma (HCC), redistribution of preoperative blood flow, and varicoceles [51,52,53,54]. TAE has become a mainstay in treating patients with aneurysms where the utmost goal is to block the blood flow through a cerebral artery, resulting in the reduction in the risk of dilation and rupture [52]. The TAE approach used to treat HCC refers to bland embolization, where the main aim is to trigger ischemic necrosis in the tumor. Some of the main advantages of TAE include its minimal risk of infection (due to its minimally invasive nature), no use of general anesthesia, quick recovery time, preservation of anatomical integrity, and high rate of success compared with other processes [55]. However, TAE has some disadvantages, such as the risk of ischemic events, user-dependent different success rates, and recurrence due to treatment failure [56]. Chemoembolization was developed in the 1980s to address these concerns. This technique is similar to TAE to treat HCC but with the addition of the localized delivery of chemotherapeutics by the same catheter [57]. Hence, this approach is known as transarterial chemoembolization (TACE) [58].

Conventional TACE or cTACE is a type of TACE that involves sequential delivery of chemotherapeutics and an ethiodized oil mixture into the tumor vasculature followed by an embolic agent [9]. In cTACE, a chemotherapeutic drug (e.g., doxorubicin) is emulsified in an oily suspension such as Lipiodol administrate, followed by gelatin sponge embolization. Contrarily, drug-eluting bead TACE or DEB-TACE is another type of TACE that contains drug-loaded microspheres and occludes the selected vessels while delivering loaded drugs in a controlled manner via a single delivery system [59]. In the past decade, both short-term and long-term outcomes of DEB-TACE have shown benefits over cTACE. A series of clinical studies demonstrated that DEB-TACE exhibited improved tolerability, a significant reduction in liver toxicity and chemotherapeutics-related side effects, and lower postprocedural pain [9,60,61]. Additionally, the DEB-TACE approach provides less complexity in handling chemotherapeutics-loaded microspheres. This approach does not require ethiodol and lowers pain and systemic toxicity in the patients. Some shortcomings of DEB-TACE are permanent occlusion (due to nondegradable microspheres), a limited selection of therapeutics to load onto the microspheres, and nontraceability of microspheres during in vivo applications [62]. The development of new degradable microspheres with the ability to load more than one therapeutic candidate may expand treatment options.

Uterine artery embolization (UAE) is another embolization technique that involves temporary occlusion of the arteries supplying the uterus by using some biocompatible microspheres to develop ischemic infarction of the fibroids [63]. In UAE studies using permanent embolic agents, it is observed that the fibroid volume can be decreased from 53.4 ± 26.9% after 3 months to 71.8 ± 26.8% after 12 months [64,65]. Different commercial embolic agents showed different rates of infarction in UAE treatment. For example, trisacryl gelatin microspheres (Embosphere^®^, Merit Medical Systems, South Jordan, UT, USA) showed better infarction rates and lower treatment failure in MRI compared with spherical PVA beads (Contour SETM, Boston Scientific) [66]. A randomized trial of 53 patients showed a significant difference (*p* < 0.025) as 8 patients treated with PVA microspheres failed the treatment (29.6%), while only one failed in the trisacryl gelatin microsphere group (3.8%) [67]. Similar results were also obtained in another study, where the UAE with trisacryl gelatin microspheres showed a superior clinical success rate over the PVA microsphere [68]. Despite the advantages of UAE in uterine fibroid treatment, concerns regarding the effect of UAE on overall fertility have resulted in recommendations against the use of UAE in women who desire to expect children in the future [50]. However, in some studies, using degradable embolics for transient occlusion via mechanical compression of the uterine arteries (up to 6 h) resulted in fibroid volume reduction and relief from menorrhagia symptoms while preserving fertility [69,70]. Such an outcome opens the door for developing degradable embolics to treat uterine fibroids efficiently without damaging fertility potential via the UAE approach.

## 3. Polymer-Based Embolic Agents

The number of polymer-based embolic materials is growing regularly based on their potential for embolization. Currently available commercial embolics, including different polymer-based particulates and liquid glues, were developed for endovascular therapies. These embolic materials are the most commonly used agents due to their various functionalities. This section discusses some of the most widely used polymeric embolics along with their clinical outcomes, benefits, and shortfalls in embolizations. Commercially available polymeric embolic agents are summarized in Table 2.

### 3.1. Particulate Embolic Agents

#### 3.1.1. Gelatin Sponge

Gelatin-based embolic agents are biodegradable and commercially available for temporary embolization. Gelatin foams are manufactured by Pfizer Inc., New York, NY, USA (i.e., Gelfoam^®^) and have been used widely in endovascular therapies since 1964 [11,71]. Gelatin foams can be used as hemostatic embolic agents to reduce subsequent blood loss during surgery. Their hemostatic ability is closely similar to that of fibrin when directly bound to the bleeding region [72]. It is reported that the coagulation time is decreased from 9.5 min to 6.2 min when gelatin powder is mixed with a whole blood sample [73]. Gelfoam sheets can be cut into small cubes or pledgets, and the particle size is typically 1 mm or more. A smaller slurry can be created from the sheets by mixing pledgets and a contrast medium such as an iodinated contrast agent for injection [74]. Another available form of gelatin sponge is powder (particle size: 40–60 μm), though it may increase the risk of ischemic tissue or neural injury through distal migration of the tiny particles [75]. Moreover, porous gelatin foam serves as a scaffold to induce cell adhesion and tissue regeneration. In general, gelatin foams have shown promising results in treating uterine fibroids, massive arterial bleeding, liver tumors, and bone malignancies [76,77]. Gelatin embolization is considered a temporary solution because of its enzymatic degradation. The gelatin-based agent is more suitable for internal iliac arteries’ embolization and occluding hepatic arteries in chemoembolization due to its degradable nature [73]. Due to its unpredictable degradability nature, gelatin-based embolization may lead to early recovery of blood flow; thus, it is not suitable for permanent occlusion.

#### 3.1.2. Spherical and Nonspherical PVA

Poly(vinyl alcohol) or PVA has been used in different morphologies for embolization treatment. Noncalibrated or nonspherical particles are prepared by mechanical fragmentation of PVA block polymer and sieve to separate particles with different size ranges from 50 to 1200 μm in the dry state [74]. PVA particles are usually mixed with diluted contrast agents to prepare a trackable suspension. PVA-based embolics were first reported in 1974 as an embolic agent in patients with vascular tumors such as liver, head and neck, and uterine cancer [78]. Since then, this embolic agent has been widely used to treat different tumors and hemorrhagic conditions such as gastrointestinal bleeding and hemoptysis. Due to the surface charges and surface hydrophobicity of PVA particles, they tend to aggregate and lead to the unintended occlusion of proximal larger vessels. Such an event is often disadvantageous when more distal embolization is required. Moreover, irregular shape and lack of size precision are other main challenges of PVA particles, which results in unpredictable embolization and catheter blockage [74]. In addition, PVA particles often adhere to the vessel wall and develop an intravascular lattice while leaving space between particles. As a result, though vessel occlusion is completed with thrombus formation among particles, recanalization can occur by capillary proliferation inside the organized thrombus.

To overcome some of the shortfalls associated with PVA particles, calibrated or spherical PVA microspheres were developed in 2003–2004 [74]. For example, Bead Block (Biocompatibles UK Ltd., Farnham, United Kingdom) is a commercially available embolic microsphere (PVA cross-linked with acrylic polymer) with size ranges of 100–300, 300–500, 500–700, 700–900, and 900–1200 μm for embolization [74]. Additionally, two other FDA-approved PVA microspheres for the embolization of hypervascular tumors and AVMs are DC Bead^®^ and LC Bead^®^ (Biocompatibles UK Ltd., Farnham, United Kingdom) and LC Bead LUMI^®^ (with intrinsic radiopacity) (Biocompatible UK Ltd.) [79]. LC Bead^®^ are PVA microspheres containing sulfonic acid groups with a negative charge. Hence, these microspheres can load positively charged antitumor therapeutics such as doxorubicin and irinotecan via an ion-exchange mechanism. Histologically, due to their lower resistance to compression, these PVA microspheres can travel more distally, and therefore are more successful in distal embolization.

**Table 2:** Summary of commercially available polymeric embolic agents for embolization.

Embolic Agents (Commercial)	Material Composition	Material Description	Therapeutic Loading	Degradability (In Vivo)	Reference
GelFoam^®^	Denatured collagen	Hemostatic gelatin sponge made of natural polymer	No	Yes	[11]
Contour^TM^	Poly(vinyl alcohol)	Irregular particles	No	No	[11]
DC Bead^®^ LC Bead^®^ Bead Block^®^	Poly(vinyl alcohol)-co-poly(2-acrylamido-2-methylpropane sulfonate)	Microsphere	Yes (Doxorubicin and Irinotecan)	No	[79,80]
HepaSphere^TM^ QuadraSphere^TM^	Poly(vinyl acetate-co-sodium acrylate)	Microsphere	Yes (Doxorubicin)	No	[81,82]
Embosphere^®^ EmboGold^®^	Tris-acyl gelatin	Microsphere EmboGold^®^ has 2% gold incorporated into the sphere	No	No	[83]
Embozene^®^	Polyphosphazene-coated polymethylmethacrylate	Microsphere	Yes (Doxorubicin and Irinotecan)	No	[84]
EmboCept^®^	Crosslinked starch	Microsphere	No	Yes	[85]
Trufill^®^	Poly(N-butyl-cyanoacrylate)	Glue	No	No	[86,87]
PHIL^TM^	Poly(lactide-co-glycolide) and Poly(hydroxyl ethyl methacrylate)	Precipitating liquid embolic	No	No	[88]
Onyx^®^	Poly(vinyl alcohol)-co-polyethylene	Precipitating liquid embolic	No	No	[89,90]

#### 3.1.3. HepaSphere^TM^/QuadraSphere^TM^

HepaSpheres^TM^ or QuadraSpheres^TM^ are microspheres composed of vinyl alcohol and sodium acrylate copolymers. In the late 1990s, these microspheres were developed and clinically used for bland embolization of HCC and peripheral AVMs [91,92]. Histologically, compared with spherical TGMS, these microspheres were able to swell and deform and conform to the vessel lumen [82]. These microspheres could swell up to 4 times in diameter within 10 min when added to a saline solution or nonionic contrast media. HepaSphere^TM^/QuadraSphere^TM^ are commercially available in dry states with the size ranges of 50–100, 100–150, and 150–200 μm, or reconstituted size ranges of 200–400, 400–600, and 600–800 μm, respectively [74]. These microspheres were approved in Europe in 2004 and in the USA in 2006. Since the microspheres are negatively charged, they can load positively charged therapeutics such as doxorubicin. The drug loading in this microsphere occurs similarly to DC Bead^®^ by an ion-exchange mechanism. These microspheres also have the potential to load noncharged drugs such as cisplatin via a reservoir effect, although the release rate of cisplatin is relatively faster within 24 h [93].

**Figure 2:**
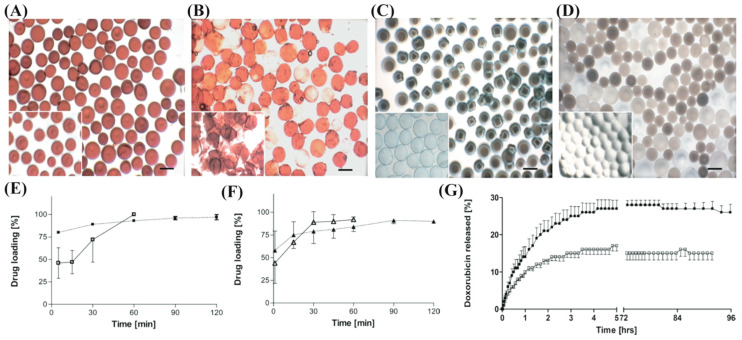
Loading and release profile of doxorubicin and irinotecan onto DC Bead^®^ and HepaSphere^TM^. Photomicrographs of doxorubicin-loaded (**A**) DC Bead^®^ and (**B**) HepaSphere^TM^. Irinotecan-loaded (**C**) DC Bead^®^ and (**D**) HepaSphere^TM^. (**E**) Loading kinetics of doxorubicin onto DC Beads^®^ (filled box) and HepaSpheres^TM^ (empty box). (**F**) Irinotecan loading profile onto DC Beads^®^ (filled triangle) and HepaSpheres^TM^ (empty triangle). (**G**) Release kinetics of doxorubicin from DC Beads^®^ (filled box) and HepaSpheres^TM^ (empty box). Reproduced with permission from Ref. [94].

Comparative studies have been conducted recently to address the differences between HepaSpheres^TM^ and DC Bead^®^. In an in vitro study, the drug loading ability, physical properties, and release profile of doxorubicin and irinotecan from HepaSphere^TM^ and DC Bead^®^ were evaluated (Figure 2A–D) [94]. During loading, DC Bead^®^ uses its ionized sulfonate groups and exchanges a water molecule for the charged drug molecule when submerged in the drug solution. In contrast, HepaSphere^TM^ utilizes mechanical loading where drug molecules are transported into the microsphere matrix via rapid water absorption. Charge interaction between the drug and the carboxylic groups holds it inside the microspheres. From the loading profile analysis, DC Bead^®^ requires 2 h for drug loading while HepaSphere^TM^ takes only 1 h to load drugs (Figure 2E,F). Both drugs showed a similar loading profile for each microsphere. However, release kinetics for both drugs exhibited different results in 0.9% saline media under 5 mL/min flow. The total percent of doxorubicin released from HepaSphere^TM^ was 18 ± 2%, while 27 ± 7% was the total for the DC Bead^®^ microspheres (Figure 2G). Moreover, the time required for both microspheres to reach 75% of the plateau value was 2.2 h. In contrast, the total percent of irinotecan released from HepaSphere^TM^ was 95 ± 9% and 102 ± 11% for the DC Bead^®^, while t_75%_ for each of the beads was 0.12 h and 1.1 h, respectively. This result indicates that the interaction between drugs and the microspheres matrices during loading occurred differently. The highly positive charge of the primary amine of doxorubicin provides stronger binding kinetics with the carboxylic groups in the HepaSphere^TM^ and the sulfonate groups in the DC Bead^®^ microspheres. Contrarily, tertiary amine in the irinotecan provides less strong binding kinetics with the respective functional groups of these two microspheres. Furthermore, differences in release kinetics also affect the distribution of drugs into the tissue of the patients.

#### 3.1.4. Embosphere^®^

Trisacryl gelatin microspheres (TGMS) are commercially available as Embosphere^®^ (Merit Medical, South Jordan, UT, USA). This material was the first commercial product made of crosslinked acrylic polymer embedded with gelatin. Commercially, these microspheres are available in different size ranges, i.e., 40–120, 100–300, 300–500, 500–700, 700–900, and 900–1200 μm. In the early 1990s, this microsphere was developed and clinically used to treat head and neck tumors and AVMs [83]. Since FDA approval in 2000, TGMS or Embosphere has been the most popular microsphere for hypervascular tumors, AVMs, and uterine fibroid embolization. Embosphere^®^ microspheres are soft and suitable for delivery via microcatheters without clogging or aggregate formation [4]. Histologically, it forms chains instead of a cluster in smaller vessels. Moreover, a single particle can completely occupy a vessel lumen. Therefore, a strong correlation was found between the sizes of the Embosphere^®^ microspheres and the diameter of occluded vessels [95].

EmboGold^®^ microspheres are commercially available TGMS particles doped with 2% gold to enhance visualization during injection [4]. It has been reported that injection with smaller microspheres (100–300 μm) resulted in marked inflammatory responses due to their deeper penetration and allogeneic overcoat [84]. In another clinical study, similar deeper penetration of smaller microspheres in meningioma embolization was also reported.

#### 3.1.5. Embozene^®^

Polyphosphazene-coated polymethylmethacrylate microspheres (Embozene^®^, Celonova Biosciences, San Antonio, TX, USA) were developed and received approval from the FDA in 2007 [96]. These microspheres are commercially available in more precise calibrated sizes with a narrower range compared with other microspheres, i.e., 40, 75, 100, 250, 400, 500, 700, 900, 1100, and 1300 μm, respectively [74]. Microspheres with different particle sizes are also incorporated with various contrast agents for particle visibility and easy size recognition. Furthermore, the unique coating of polyphosphazene (commercial name: Polyzene-F^®^) act as an antithrombogenic and anti-inflammatory material [96]. The properties of Polyzene-F^®^ help it persist for a long time in patients with benign conditions such as uterine fibroid or meningioma. This microsphere does not have biodegradability, which hinders its application in temporary embolization, e.g., to treat hemorrhage.

#### 3.1.6. Other Degradable Microspheres

Degradable starch microspheres are composed of crosslinked hydrolyzed starch and amilomeres and were developed in the mid-1970s by Pharmacia AB to provide a highly transient embolic effect [9]. This microsphere is available under two commercial names, i.e., EmboCept^®^ S by PharmaCept and Spherex^®^ by Magle Life Sciences [97]. Although the material half-life is around 40 min per manufacturer, in practice, this range is between 25 and 60 min [9,85]. Both commercially available DSMs are 50 μm in size, hence, intended for the temporary embolization of small vessels. DSMs have been clinically practiced for cTACE to treat HCC by providing transient occlusion to decrease blood flow in the tumor bed. This material also helps to enhance the time to keep chemotherapeutic emulsion in the cancerous tissue. DSMs have been recommended for use in patients who need multiple chemotherapeutic treatments by cTACE [98]. This material also has been used in patients with a reduced hepatic function who cannot endure more damage to the surrounding hepatic tissue induced by ischemic necrosis [99].

**Figure 3:**
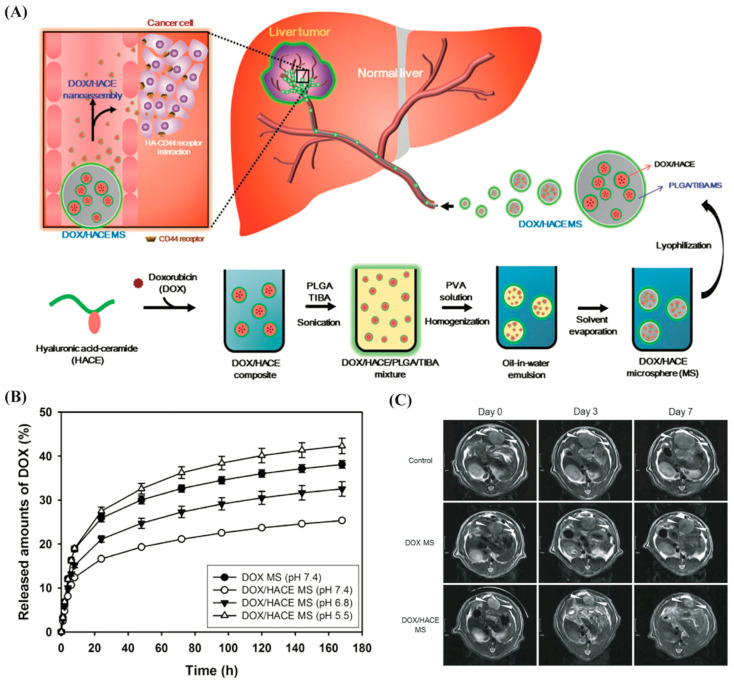
Doxorubicin (DOX)-loaded hyaluronic acid–ceramide (HACE) nanoassembly-releasing PLGA microspheres for TACE therapy of liver tumor. (**A**) Schematic outline of the DOX/HACE microsphere fabrication. (**B**) Drug release profiles from different microspheres at different pH (i.e., pH for DOX is 7.4, and pH for DOX/HACE are 7.4, 6.8, and 5.5). (**C**) Series of MR images of liver tumors in control (sham operation), DOX microspheres, and DOX/HACE microsphere groups on day 0 (pre-treatment), 3, and 7 after intra-arterial administration. Reproduced with permission from Ref. [100].

Some other degradable polymeric particulates such as hyaluronic acid and poly(lactic-co-glycolic acid) (PLGA) have been designed for embolization application. For instance, a PLGA microsphere has been developed by entrapping the doxorubicin (DOX) and hyaluronic acid–ceramide (HACE) in PLGA (Figure 3A) [100]. The DOX/HACE nanoassembly is released from the microspheres after being administered into the hepatic artery. A better drug release profile was observed for DOX/HACE microsphere group over the DOX microsphere in the acidic environment (i.e., tumor-specific region) (Figure 3B). The cellular internalization efficiency of the therapeutic was higher for the DOX/HACE microsphere in liver tumor cells (HepG2 and McA-RH7777 cells) over the control group (DOX microsphere only). Interestingly, the elevation in the cellular accumulation of DOX and its better anticancer performance was observed in DOX/HACE-based nanoassembly released from the DOX/HACE microspheres (Figure 3C). Such microspheres can be used as a therapeutic agent-loaded hyaluronic acid nanoassembly-releasing microsphere system for HCC embolization application.

### 3.2. Liquid Embolic Agents

Liquid embolic materials can flow through the vessels, which allows them to deeply penetrate the smaller vessels. Once they reach the target vessel, they become solidified to occlude vasculature. Unlike particulate embolics, liquid embolic agents can be used to occlude vasculature of a wide range of diameters.

#### 3.2.1. N-Butyl Cyanoacrylate (NBCA)

Embolic glue, N-butyl cyanoacrylate was approved by the FDA in 2000 to treat cerebral arteriovenous malformations [101]. NBCA is a clear liquid in its monomer state, but once it encounters ionic substances such as blood, saline, or ionic contrast media, it polymerizes to form a stiff matrix. Primarily, NBCA is recommended for AVM embolization, acute bleeding in the gastrointestinal tract, or preoperative embolization for hepatectomy (partial) [102]. The adhesive nature of NBCA allows this material to be used in coagulopathic patients, while other embolic materials such as coils or Gelfoam^®^ are not recommended due to their high chance of occlusive thrombus formation within the vessels [103,104]. NBCA embolic material mechanically occupies the intravascular lumen via adhesion and halts the blood flow in patients. The adhesive nature of NBCA is also a limitation in some cases where multiple injections are required [105]. NBCA may adhere to the catheter and occlude the lumen in a case where the subsequent injection is crucial. Trufill^®^ is a commercial ‘ready-to-use’ glue that contains NBCA, ethiodized oil, and tantalum powder. Depending on the flow properties and the anatomy of target vessels, NBCA concentration ranges between 25 and 67% in the glue [101]. When NBCA combines with ethiodized oil in blood, polymerization slows down from fewer than 1 s at 67% NBCA to 6 s at 25% NBCA. One of the main limitations of NBCA is that it releases formaldehyde upon polymerization, resulting in the inflammation of the vessel wall and surrounding tissue and sometimes causes chronic granulomatous inflammation.

#### 3.2.2. Precipitating Hydrophobic Injectable Liquid (PHIL^TM^)

A recently developed injectable liquid embolic is PHIL^TM^ (manufactured by MicroVention Inc., Aliso Viejo, CA, USA), which has been approved in Europe for clinical use in the embolization of lesions in arteriovenous malformations, hypervascular tumours, and other peripherals and neurovasculature [88]. PHIL^TM^ is a nonadhesive embolic agent consisting of poly(lactide-co-glycolide) and poly(hydroxyl ethyl methacrylate) copolymers. A contrast agent (i.e., triiodophenol) is also incorporated into the monomers by covalent bonding to provide radiopacity for visualization [106]. Initially, PHIL^TM^ is suspended in dimethyl sulfoxide (DMSO) until it encounters body fluids such as blood or water. Once it reaches there, polymers precipitate into a solid and block the vasculature instead of forming layers. Commercially, three concentrations of PHIL^TM^ are available, i.e., 25%, 30%, and 35% (concentration of polymer by weight) [9]. One of the major distinguishable features of PHIL^TM^ is that no microcatheter occlusion occurs during the injection. In addition, this embolic offers less resistance to injection, hence, allowing for deeper penetration. This lower resistance often increases the risk of overpenetration into the venous drainage. Clinical data for PHIL^TM^ to treat dural arteriovenous fistulas (DAVFs) showed that the enhanced venous penetration of PHIL^TM^ was advantageous to filling fistula feeders and provided better outcomes [107]. Another clinical study reported the successful use of PHIL^TM^ to treat ruptured plexiform AVMs [108].

#### 3.2.3. Onyx^®^

Onyx^®^ is another potential liquid embolic agent made by Medtronic plc. for the embolization of intracranial AVMs in the 1990s [109]. This embolic liquid is made of ethylene–vinyl alcohol (EVOH) copolymers dissolved in DMSO. Once the DMSO dissipates within the bloodstream, Onyx^®^ solidifies in an ‘outside-in’ fashion due to the formation of solid EVOH precipitates [90]. As a result, an instantaneous solid cast forms around the exterior while the interior of the flow remains fluid, enabling it to flow deep into the lesion. Onyx^®^ formulations with lower concentrations of EVOH have low viscosity, thus travelling more distally from the catheter tip [110]. Contrarily, highly viscous Onyx^®^ formulations provide better control in a high-flow environment. Despite their different formulation, they completely solidify within 5 min after injection [111]. In addition to treating AVMs, Onyx^®^ has also been applied successfully to treat gastrointestinal and cardiopulmonary hemorrhages, bleeding from aneurysms, preoperative vascular tumors embolization, and peripheral arteriovenous malformations [111]. Onyx^®^ is a permanent occlusive material that remains stable up to 5.25 years after occlusion. The use of Onyx^®^ requires special attention in using DMSO-compatible catheters and flushing the catheters with DMSO before administration. Although Onyx^®^ is nonadhesive, the catheter may still become clogged if the long plug (>2 cm) forms around the tip during injection [9].

The liquid nature of Orynx^®^, PHIL^TM^, and NBCA allows them to pass through smaller vessels, which is advantageous over particulate embolics. For example, Onyx^®^ can occlude vessels of 5 μm in diameter while NBCA can occlude up to 20 μm in diameter [112]. Despite this advantage, NBCA has a quick working time because of the rapid polymerization upon contact with blood and adhesive to vessel walls and the delivery system [113]. As a result, permanent catheter clogging occurs, which requires delivery system replacement, thus extending the working time. In contrast, Onyx^®^ is nonadhesive and has a longer working time. However, if Onyx^®^ is injected rapidly, it may cause vascular inflammation and angionecrosis due to the high concentrations of DMSO at the catheter tip [97,114]. In a study of 32 patients (22 patients with Onyx^®^ and 10 with NBCA), Natarajan et al. analyzed the histopathological data of resected AVMs after embolization [112]. They found perivascular inflammatory damage among 90.9% of the Onyx^®^ and 90% of the NBCA tissue samples. This inflammatory damage could be explained as a response to the foreign material in the vessel lumen. Moreover, 60% of Onyx^®^-injected vessels showed angionecrosis, while 40% of the NBCA-injected vessels had angionecrosis. In another study, PHIL^TM^ was used alone and combinedly with Onyx^®^ to treat AVMs [115]. It was found that PHIL^TM^ exhibited better outcomes over Onyx^®^ in terms of ease of use, quick plug formation, and lower imaging artifact at follow-up after treatment. Besides, histopathological data indicated that PHIL^TM^ caused moderate vascular inflammatory damage while Onyx^®^ had lower inflammation. Therefore, all these studies reflected favorable outcomes for PHIL^TM^ as a new and superior liquid embolic agent to treat disease conditions.

Finally, these results reflect the complications with these materials that need to be addressed to make outstanding next-generation liquid embolics in future embolization.

### 3.3. Emerging Embolic Agents

Currently, a few embolic agents have received regulatory approval, and some of them are commercially available. Most liquid embolics have the potential for use in endovascular embolization. However, these materials have some pitfalls such as lack of biocompatibility and degradability. Furthermore, the polymer-based liquid embolic materials exhibited lower cell viability over nontreated cell cultures (fibroblast cell lines). Higher pH conditions that require chemical crosslinking are supposed to be partially responsible for lowering cell viability in a liquid gelling system. To overcome all these issues, emerging polymer-based injectable embolics are explored in the following subsections.

#### 3.3.1. Thermoresponsive Embolic Gels

**Figure 4:**
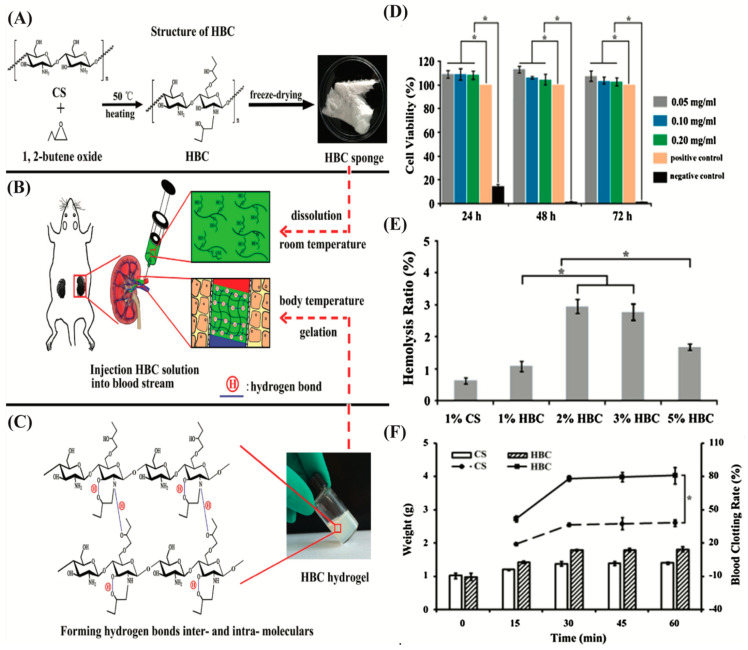
(**A**) Schematic illustration of HBC synthesis. (**B**) Injection of HBC solution into a rat renal artery model. (**C**) inter and/or intrahydrogen bond formation in HBC hydrogel. (**D**) cytotoxicity analysis of HBC hydrogel by MIT assay. (**E**) The effects of HBC solution on in vitro hemolysis study. (**F**) Blood clotting rates of HBC hydrogel and chitosan (CS) hydrogel. *: *p* < 0.05, statistically difference. Reproduced with permission from Ref. [117].

Temperature-sensitive gelling systems have been developed recently to reduce cytotoxicity and gelation time. In a study, an injectable chemical and physical crosslinked N-isopropyl acrylamide (NIPAAm)-based gels for endovascular embolization [116]. These hydrogels are composed of the copolymers of NIPAAm and hydroxyethyl methacrylate (HEMA) with the functionalization of pentaerythritol tetrakis 3-mercapto propionate and olefins. This gelling system formed elastomeric hydrogels at pH 7.4. The cytotoxicity of this poly(NIPAAm-co-HEMA-acrylate) injectable gels was assessed using fibroblast by direct and indirect methods. Even though this material was not cytotoxic, it inhibited cell adhesion and proliferation on the gel surface. In another study, thermosensitive hydroxybutyl chitosan (HBC) hydrogel was developed for vascular occlusion (Figure 4) [117]. Once this hydrogel was injected into the renal artery of a rat, a fast sol–gel transition converted the HBC solution into a hydrogel and tightly adhered to the inner wall of the blood vessel. As a result, the blood vessel was occluded immediately. Li et al. developed a thermoresponsive NIPAAm-N-propylacrylamide (NPAAm)-vinyl pyrrolidone (VP) terpolymers (PNINAVP) hydrogel for embolization application [118]. They added Iohexol (a radiopaque agent) to make it trackable for follow-up imaging. The reversible sol–gel transition occurred quickly within 1 min in response to temperature change. Then, the hydrogel solution was injected into the rete mirabiles (RM) of six swine. Angiographical data obtained immediately after the injection exhibited a complete occlusion of the RM, while no recanalization was reported in follow-up imaging after one month of operation. Moreover, histological results show no acute inflammatory damage inside the RM and the surrounding tissue of the animals.

Chitosan has a high adhesion ability to a blood vessel but forms precipitation at pH 6.2, which often hinders its potential application for TAE [119]. To overcome this issue, a liquid embolic agent made of 2% chitosan and 56% b-glycerophosphate disodium salt (b-GP) was prepared for embolization [120]. This embolic hydrogel can be flowable in the physiological pH at low temperature and turn into a hydrogel at 37 °C in a thermoresponsive manner. This liquid embolic agent blocked the rabbit’s renal artery within eight weeks of treatment without any recanalization. A modified trackable hydrogel with the same composition of chitosan and b-GP was developed with an X-ray contrast reagent (i.e., Visipaque) for acute embolization of porcine spleen and gastric vessels [121]. Poloxamer 407 or poly(ethylene glycol)-poly(propylene glycol)-poly(ethylene glycol) is a thermoresponsive triblock copolymer that shows sol–gel transition upon temperature changes from 4 °C to 37 °C [122]. This material has been applied as a temporary embolic agent for the embolization of the arteries of animals. The results show successful embolization in the animal arteries while it caused recanalization of the vessels after some time. A composite hydrogel (PSHI-Ca^2+^) was developed consisting of poloxamer 407, hydroxymethyl cellulose, sodium alginate, iodixanol, and Ca^2+^ to solve this issue [123]. The alginate portion in this hydrogel enhances the retention rate of Ca^2+^ ions and prevents the erosion of the hydrogel by forming a stable three-dimensional structure in the water environment. This PSHI-Ca^2+^ hydrogel showed an excellent curative effect in VX2 liver tumors in rabbits by effectively blocking vessels around the tumor within 7 days of treatment.

Although these temperature-sensitive hydrogels show potential in embolization, still, there is room to strengthen their mechanical properties. Such improvement will make them a suitable candidate for longer embolization as well.

#### 3.3.2. pH-Responsive Gelling System

**Figure 5:**
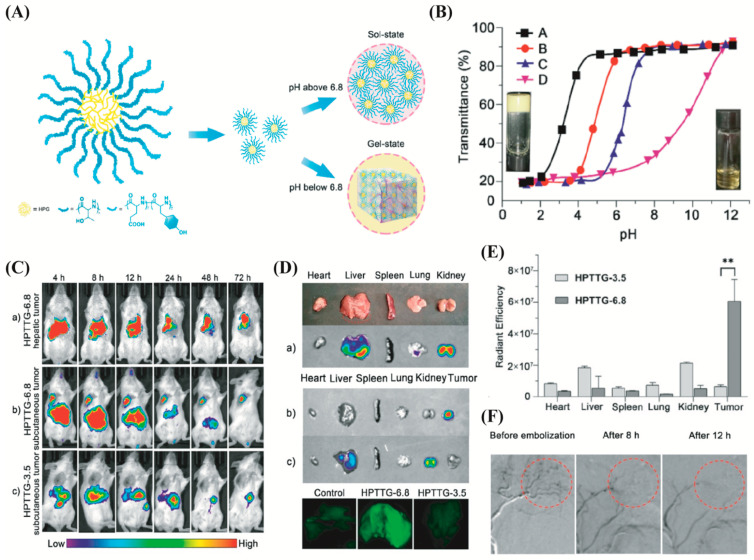
(**A**) Schematic outline of the gelation mechanism of HPTTG. (**B**) Transmittance-pH curve of HPTTG-based copolymers to analyze the sol–gel transition. (**C**) Efficient accumulation in tumors by doing fluorescence imaging at 4, 8, 12, 24, 48, and 72 h after post injection. (**D**) Fluorescence imaging of hepatic tumor and subcutaneous tumor of mouse tissues after 72 h postinjection (a: HPTTG-6.8 (hepatic tumor); b: HPTTG-6.8 (subcutaneous tumor); c: HPTTG-3.5 (subcutaneous tumor)). (**E**) Quantification of two HPTTG microspheres biodistribution in right forelimb tumor model. (**F**) DSA images of the mouse hepatic tumor before embolization, after 8 h, and after 12 h of embolization. **: *p* < 0.01, statistically difference. Reproduced with permission from Ref. [129].

Although thermoresponsive hydrogel has the potential for embolization, early gel formation due to quick temperature change during injection is still a prime concern that needs attention [124]. Other physiological factors such as pH could be a salient parameter to design embolic hydrogels. In response to the changes in environmental pH, the pH-responsive hydrogel changes from a hydrophilic state to a hydrophilic state. pH-responsive hydrogels containing therapeutic agents could be transferred as a liquid at a controlled pH through a catheter and form gelation once it reaches the target lesion pH and subsequently releases the therapeutics [125]. Sulfamethazine (SM)-based hydrogels are typical examples of pH-responsive hydrogels that have been developed to treat vascular intervention therapy. A few examples of this kind of hydrogel are polyethylene glycol (PEG) and polyurethane sulphide sulfamethazine (PUSSM) copolymer or PEG-PUSSM, poly(e-caprolactone) (PCL), SM, PEG copolymer (PCL-PEG-SM), and the triblock copolymers made of poly(e-caprolactone-co-lactide)-poly(ethylene glycol)- poly(e-caprolactone-co-lactide) (PCLA-PEG-PCLA) and SM [126,127,128]. Sulfadimethazine-based pH-responsive hydrogels are ionized (SM part) and hydrophilic in nature at high pH, while at physiological pH, the SM portion of the hydrogels become deionized and hydrophobic. This means that the decrease in pH triggers the sol-to-gel transition and formation of a 3D gel network. Another pH-responsive hydrogel is hyperbranched poly(amino acid) (HPTTG), developed for noninvasive target embolization (Figure 5A) [129]. The sol–gel transition with reducing pH of HPTTG was regulated by adjusting the acidic amino acids in copolymers (Figure 5B). The accumulation of HPTTG is mainly due to the acidic environment of tumors that triggers sol–gel transition (Figure 5C). Despite using this polymer as an embolic for embolization of unresectable hypervascular tumors, it can be used combinedly with controlled-release, thermal ablation, trackability, and synergistic therapy. It has been reported that the unique pH-responsive property helps to achieve good performance in rabbit liver embolization and renal vasculature embolization [126].

#### 3.3.3. Self-Healing Embolic Gels

Self-healing hydrogels show autorepair ability under physiological conditions. Hence, these hydrogels exhibit their potential as an embolization material. For example, a self-healing hydrogel made of glycol chitosan, dibenzaldehyde-terminated PEG, and carbazochrome was prepared for chemoembolization [130]. In this hydrogel, Schiff-base bonding helps self-healing by uncoupling and recoupling imine linkages. The gelation of this hydrogel occurred within 3 min, while Carbazochrome was released from the hydrogel continuously for 6 h as a hemostatic agent to block blood flow. This hydrogel was developed initially for in vivo renal artery chemoembolization in a rat model.

#### 3.3.4. Shear-Thinning Hydrogels

In recent years, shear-thinning hydrogels have drawn great attention in endovascular embolization. These hydrogels are self-healable, easily injectable, and capable of delivering therapeutic agents and growth factors [131]. They are ideal for facilitating injection during transcatheter delivery due to decreased viscosity at an enhanced strain rate. For instance, a shear-thinning biomaterial (STB) was developed using gelatin (type A) and silicate nanoplatelets (Laponite^TM^ XLG) to use as a hemostatic agent for embolization [132,133]. The positively charged gelatin interacts with silicate due to anisotropic charge distribution. As a result, they self-assemble, form dynamically, and show the shear-thinning property. Furthermore, silicate nanoplatelets trigger coagulation by concentrating clotting factors. The STB shows hemostatic behavior due to the intrinsic coagulation of gelatin and silicate nanoplatelets. STBs exhibited excellent results in arterial embolization in small and large animals. This material blocked the blood flow immediately after being injected into a pig’s left external iliac artery. A pig’s iliac artery has a high flow rate of 100 cm s^−1^ like human iliac arteries. Hence, STB shows the potential to provide stable and complete occlusion.

#### 3.3.5. Other Potential Polymeric Embolic Agents

Besides the above-mentioned established polymer-based emerging embolic agents, some new polymeric embolics have also been developed in recent years. For instance, the PPODA–QT polymer composite was developed in the basic environment (NaOH) from liquid organic monomers poly(propylene glycol) diacrylate (PPODA) and pentaerythritol tetrakis 3-mercapto propionate (QT) [134]. This composite material transforms into gels to form a network through crosslinking by a Michael-type addition in a time-dependent manner. The mixture can be delivered through a catheter before being transformed into fully crosslinked gels. Furthermore, high pH, presence of surfactant, and premixing time all trigger faster gelation kinetics of the PPODA–QT system. The type of contrast agent used in the system also alters the gelation kinetics. For example, rapid gelation occurs when the Conray contrast medium (Mallinckrodt, St Louis, MO, USA) is used, while the Omnipaque system slightly slows down the gelation kinetics of the PPODA–QT system [135]. There is a risk of uncontrollable and unpredictable swelling after embolization because it possibly overstresses weak blood vessels. Additionally, experimental evidence suggests that its biocompatibility and long-term stability make this system a potential candidate for embolization treatment (e.g., aneurysm). In a study, a new injectable embolic gel (PCLA-PUSSM) was developed with radiopacity property using poly(e-caprolactone-co-lactide) (PCLA), poly(urethane sulfide sulfamethazine) (PUSSM), and poly(ethylene glycol) (PEG) [128]. The gelation conditions (temperature/pH) of this hydrogel were 25 °C/8.5 (sol) to 37 °C/6.6 (gel), respectively. An in vivo experiment showed that the 25 wt% PCLA-PUSSM exhibited successful gelation at the target site in a rabbit hepatic tumor model. No significant washout of the polymers into the bloodstream was noticed in the test. Such dual responsive injectable radiopaque polymer gels may have the potential to be used for chemoembolization targeting unresectable hepatic carcinoma, cerebral aneurysms, and other diseases. In another study, Lym et al. developed a pH-responsive PCL–PEG–SM copolymer that experienced a sol–gel transition from pH 8.0 to pH 7.4 at 37 °C [127]. This copolymer solution could be intra-arterially administrated in a rabbit VX2 liver tumor model at pH 8.0 for embolic application. Huynh et al. developed a dual responsive hydrogel made of poly(amino ester urethane) (PAEU) block copolymer that demonstrated its potential as a stimuli-responsive embolic agent in TAE application [136].

Besides embolics with temperature and pH responsiveness or made from chemical crosslinking approaches, hydrogels for embolization might also be made from electrostatic interaction. For instance, a calcium-alginate crosslinked network has been developed in this process for vascular embolization. Furthermore, decellularized tissue and a series of nanoclays (i.e., Laponite nanoclay and gelatin and silicate nanoclay) might be polymerized into hydrogels via electrostatic action to make embolics with good shear-thinning properties.

### 3.4. Embolic Agents with Trackability

The design of an embolic material with an incorporated imageable or radiopaque agent capable of carrying therapeutics would provide accurate spatial localization of the embolic and the therapeutics. Embolic agents with such trackability would enable us to better understand the in vivo embolization and give a real-time visualization regarding the location and the level of occlusion. Intrinsic radiopacity can be added to microspheres via adding the radiopaque moiety onto the core polymer or using radiopaque monomers during polymerization. For trackable embolization, iodide is one of the most commonly used radiopaque agents that could be trapped easily in hydrogels [137]. Sharma et al. developed a PVA-based hydrogel microspheres loaded with Lipiodol^®^ and analyzed the iodine content followed by a test in saline solution to assess Lipiodol^®^ stability and release [138]. The microspheres trapped up to 35.7% iodine by weight while the cumulate release of Lipiodol^®^ was below 20% during a stability test over a 27 h period. The radiopacity of these PVA microspheres was later validated with routine fluoroscopy and CT. During in vivo visualization in normal swine liver, it was found that these microspheres were not visible during the injection; however, once they accumulated within the hepatic artery, they were visualized by fluoroscopy or CT. In a study, Zhao et al. developed thermoresponsive poly(N-isopropylacrylamide-co-butyl methyl acrylate) (PIB) hydrogels with an iohexol contrast agent for visualization in embolization [139]. They found that iodinated compounds significantly affected the sol–gel behavior of the hydrogels. Additionally, using this embolic increased the difficulty and risk of operation. It is worth mentioning that the iodide-loaded PIB showed poor long-term imaging performance as it might be rapidly metabolized in the body. Therefore, Ma et al. replaced iodide with gold nanoparticles as a contrast agent [140]. Gold nanoparticles are superior to iodide because it gives better imaging performance in X-ray. Additionally, they have better imaging time and biocompatibility. Polymer embolics containing gold nanoparticles showed higher angiographic capabilities and better vascular embolization effects in the animal study. In a study, Horák et al. designed a polymeric hydrogel with intrinsically radiopaque beads, which are porous (important for effective embolization) and visible by X-ray imaging [141]. Commercially available LC Bead LUMI^TM^ microspheres are intrinsically radiopaque and currently used in the management of HCC [142]. Aliberti et al. reported that this microsphere during the treatment of HCC demonstrated low levels of toxicity and some adverse events, although it provided real-time direct visualization of the microspheres [143]. Liquid metal is a low melting point (near room temperature) metal with the features of radiopacity, low viscosity, and fluidity to enhance its injectibility. Fan et al. prepared radiopaque hydrogels by rapid crosslinking between liquid metal/calcium alginate mixture and calcium chloride solution [144]. This hydrogel has advantages in X-ray imaging over currently available commercial contrast agents due to its high density. In vivo tests in mouse blood vessels also confirmed its efficacy and safety after nearly a month of monitoring. During one month of monitoring, no inflammation or infection was reported. These materials had been applied to embolize rabbit ear tumours by blocking the middle artery blood flow towards the tumor. The tumor was successfully suppressed in two weeks. Moreover, polymeric carbon nitride (CN)-based materials can also serve as luminescent probes to provide real-time visualization of the embolization stages [145].

**Figure 6:**
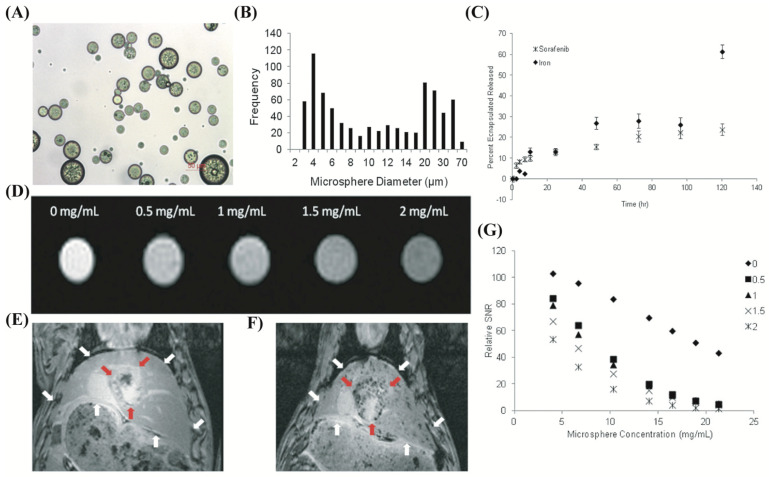
(**A**) Confocal microscopic image of PLG microspheres with sorafenib and iron oxide (scale bar represents 50 μm). (**B**) Histogram of microsphere sizes (average size diameter was 13 μm). (**C**) Release kinetics of sorafenib and iron oxide from the microspheres. (**D**) MRI image (T_2_^*^ weighted) of PLG sorafenib iron oxide agar phantoms. The decay rates increased (signal dephasing effects of iron oxide) with increasing microsphere concentration. (**E**) Preprocedural MRI image (T_2_^*^ weighted) of the liver (white arrows) and tumor position (red arrows). (**F**) Postprocedural MRI image (T_2_^*^ weighted) of the liver demonstrates intense signal loss at the tumor due to the catheter-directed PLG microspheres delivery. (**G**) Signal decay rates with respect to increased microsphere concentration. Reproduced with permission from Ref. [146].

Embolic microspheres with the capability of drug carrying and MRI visibility using iron oxide nanoparticle were developed recently (Figure 6). Poly(lactide-co-glycolide) PLG-based microspheres encapsulated with the ferrofluid of iron oxide nanoparticles (IONP) were developed and loaded with sorafenib (FDA approved to treat hepatocellular carcinoma) (Figure 6A) [146]. These microspheres are spherical with a lower size (average diameter: 13 μm) than commercial microspheres (Figure 6B). An in vivo test in a rat HCC cell line showed that these microspheres significantly reduced cell proliferation while successfully being detected by MRI (Figure 6D). The visibility and therapeutic effectiveness of these microspheres in an in vivo test (rat HCC model) showed that although the clinical response was not significant compared with the control group, they accomplished the goal of in vivo tracking.

Despite these commonly used contrast agents, some other radiopaque agents were introduced recently, such as barium sulphate and silver iodide complexes for radiopacity [147,148]. Moreover, tantalum-containing polyurethane and barium sulphate-containing alginate microspheres were also tested for radiopacity and it was found that the organoiodine compound is still the most effective radiopaque agent [149,150]. For instance, Wang et al. developed barium sulphate-containing alginate microspheres using a microfluidic system for CT imaging [149]. These microspheres were polydisperse and large in size (diameter ranges between 149 to 380 μm). Microspheres with different weight percentages of barium sulphate were tested for CT visibility. The highest barium sulphate content achieved was 9% from their study, although a minimum of 5 wt% was required for CT visibility. Despite the visibility of these microspheres, they could easily be ruptured and lead to uneven-shaped microspheres.

The current literature demonstrates that the trackable polymeric embolics need further improvement before being ready to use in embolization applications. Some key features such as rheological properties need to be verified and compared with commercial products. The size ranges of the fabricated microspheres are still too narrow to prevent nontarget embolization and have adverse effects. Finally, more thorough efficacy studies and long-term biocompatibility tests are needed to confirm their long-term stability and the ability to treat vascular malformations and tumors such as liver HCC.

## 4. Injectable Polymers for Cell Delivery

Stem cell transplantation or cell therapy has the potential to enhance the healing and regeneration of damaged tissue. A wide range of promising investigations of cell therapy approaches has been conducted previously to treat vascular malformations such as aneurysms [151]. Different cell types, such as endothelial progenitor cells (EPCs), mesenchymal stem cells (MSCs), and vascular smooth muscle cells (VSMCs) have been suggested as target cell therapies for damaged tissue repair [152]. Among them, MSCs have great potential, as they can migrate to damaged tissues and induce regeneration by secreting bioactive factors, inhibiting inflammation, and recruiting progenitors to differentiate and replace damaged cells [153]. These advantages of MSCs indicate the notion of combining embolization with cell therapy to enhance regenerative mechanisms. As a result, treatment efficacy will improve regarding occlusion, durability, and safety. Successful cell therapy also requires improvement in the function of transplanted cells. Therefore, polymeric hydrogels can be designed to support therapeutic cell transplantation. Properties such as injectability, rapid gelation in vivo, mechanical property, and vascularization are the main features of hydrogels to support cell transplantation [154]. These properties can be achieved by designing hydrogels with shear thinning, stimuli responsiveness, controlled porosity, immobilization of natural ligands, and native extracellular matrix (ECM) composition.

At present, low-viscosity fluids such as saline have been used for topical cell transplantation, which results in substantial cell death (up to 40% cell viability loss) during the injection of cells via a syringe needle [155]. During cell transplantation, no carriers (e.g., hydrogel or other material) are used other than saline in most clinical interventions. Therefore, the development of cell transplantation materials has the potential for successful cell therapy applications.

Natural materials (e.g., collagen) can be derived from ECM and purified to keep inherent bioactivity for cell transplantation [156]. In contrast, synthetic polymer materials offer broad design space to provide appropriate physical behavior and offer the potential to assimilate defined bioactive elements for cell transplantation. Both synthetic and natural injectable polymer materials can provide an environment like a cellular environment to improve the success of cell therapy. These polymer materials provide a temporary niche for delivered cells and a convivial microenvironment for cell migration, proliferation, and the exchange of bioactive compounds. Furthermore, the interconnected porous polymeric network can play a crucial role in overall cell survival, migration, proliferation, penetration of metabolic compounds, and angiogenesis [157]. For example, a porogen-free injectable monodispersed microgel particle was developed and showed successful cell transplantation to treat chronic wound healing [158]. This microporous gel particle system was composed of multiarmed poly(ethylene)glycol-vinyl sulphone, arginyl-glycylaspartic acid (RGD), and transglutaminase peptide substrate. These components were assembled via the microfluidic water-in-oil emulsion technique into microporous gel particles. These particles were then injected into a mold using a syringe and formed annealed particles by noncanonical amide linkages mediated via activated factor XIII. In another study, an injectable polymeric hydrogel was designed using RGD-functionalized PEG-b-PLA and hydrophobic lipid dodecyl chains with modified hydroxypropylmethylcellulose [159]. This injectable hydrogel was used to deliver human MSCs (hMSCs) and showed improved transplantation efficacy and extended local retention of hMSCs for up to 2 weeks.

Previous studies reported that the injectable polymer gels can regulate the secretion of paracrine factors from stem cells by controlling physicochemical features such as stiffness, oxygen tension, multiaxial tension, and bioactive molecules [160]. The paracrine factors are different growth factors, chemokines, cytokines, and bioactive lipids. They are released from activated cells such as hematopoietic stem cells, adipose-derived stem cells, cardiac stem cells, or neural stem cells. These factors can regulate cell biology while maintaining interaction with the microenvironment.

The application of injectable polymer hydrogels in cell therapy is promising. Currently, much research is ongoing to develop a new hydrogel carrier for cell delivery. Improved delivery and retention of functional cells at delivery sites are two primary designing criteria to develop polymeric cell carriers for cell therapy. The implementation of these designing criteria can create an effective injectable polymer material for embolization while serving as a carrier for cell delivery which will open the potential for effective regenerative therapies.

## 5. Conclusions and Future Outlook

The future for polymer research focused on embolization agent development holds great promise. In the last few decades, different polymeric materials have been designed and modified to develop a variety of embolic agents for the treatment of various vascular malformations and hypervascular tumors such as hepatocellular carcinoma and uterine fibroids. The improvement in the material properties (e.g., from irregularly shaped particulates to precisely calibrated microspheres) has ameliorated clinical outcomes. So far, stimuli-responsive hydrogels have not been approved for embolization applications, but these materials have attracted much attention due to their unique physicochemical properties. Moreover, the inherent trackability of the embolic agents can provide both visualizations during delivery and monitoring embolization response for a long time. Though immense advancement has been made in developing next-generation embolic agents for embolization, still, there is room for improvement. The next-generation embolics development depends on the understanding of the material properties. For example, physical and rheological properties are crucial to predicting the occlusion level and tissue/embolics interactions. So far, no standardized protocols have been developed to compare different agents. It is important to assess the biocompatibility, cost effectiveness, and ease of deployment to determine the best embolic agent for application. The ideal embolic agent should be able to block the artery and fill 100% of the vasculature quickly, carry therapeutics, provide intrinsic visibility, prevent recanalization and fragmentation, and possess clinically relevant shelf life. Therefore, it is essential to assess both short and long-term stability and biocompatibility of the embolic agents. In addition to these features, retrievability and reversibility are also important, although these properties have not been adequately addressed yet. It is worth mentioning that the development of reversible embolic material would offer flexibility to benefit embolization. Furthermore, DEBs have been designed and clinically used, but they cannot penetrate arteriocapillaries due to finite sizes. Alternatively, liquid embolic agents have been designed as a promising system to penetrate deeply into all branches (blood vessels) of the tumor. Although enormous efforts have been devoted to developing polymer microspheres with biodegradability and sustained therapeutic release ability, a specific system designed and tested for embolization is still limited.

Real-time imaging is a fundamental tool in practicing interventional radiology, particularly during surgical procedures to treat vascular malformations. So far, the injection of a contrast agent is a common practice in this field; however, it does not satisfactorily provide feedback about the location of the embolic within the vessels. Therefore, trackable polymer microspheres have the potential to give real-time feedback to the physician by providing the location and distribution of the embolics and therapeutics within cancerous tissues. Despite the potential of such trackable polymeric embolics, the development of such materials is still in the infancy stage.

Currently, no FDA-approved cell-carrying embolic agents are available for clinical use. Although some preclinical studies are ongoing, such as coil-mediated endovascular delivery of cells for aneurysms treatment, no injectable polymer embolics for cell delivery have been reported yet [161,162]. Therefore, the potential for cells carrying trackable embolic materials for endovascular embolization needs broader evaluations. Furthermore, controlling physicochemical properties such as porosity and gelation are a few key parameters that need to be optimized for future injectable polymeric cell delivery systems. Injectable polymer hydrogels with these optimum properties will enhance encapsulated cell attachment, migration, and differentiation. One way to control physicochemical properties (e.g., porosity) is by mixing biodegradable microbeads into the hydrogel; this in turn also increases cell migration and retention. However, improper leakage of microbeads may cause unintended occlusion in nontargeted vessels, resulting in ischemic tissue damage. Therefore, more care is needed to achieve interconnected controllable porosity in such hydrogel. Moreover, the cell-carrying hydrogels need to meet the appropriate mechanical needs for successful stem cell transplantation in all three stages, i.e., injection, postinjection, and long-term survival [155]. An ideal hydrogel candidate would be the one that can alter its mechanical features in different stages of cell transplantation for a variety of endovascular embolization applications.

The tuning of material properties allows for the development of diverse polymeric embolic materials. The designing and development of embolic agents will continue to evolve as more clinical data renders insight into the issues and other unmet needs. Future directions in developing multifunction polymeric embolics will need to focus on some key design criteria such as enhanced biocompatibility, degradability, trackability, intrinsic bioactivity, and various therapeutic (e.g., cells or drugs) loading ability. This review has sought to outline this fertile and promising field which requires an integrated effort from all disciplines to revolutionize next-generation embolics for endovascular therapies.

## Data Availability

Not applicable.
